# The value of machine learning in preoperative identification of lymph node metastasis status in endometrial cancer: a systematic review and meta-analysis

**DOI:** 10.3389/fonc.2023.1289050

**Published:** 2023-12-20

**Authors:** Zhonglian Ren, Banghong Chen, Changying Hong, Jiaying Yuan, Junying Deng, Yan Chen, Jionglin Ye, Yanqin Li

**Affiliations:** ^1^Department of Obstetrics and Gynecology, Chengdu Shuangliu Distract Maternal and Child Health Hospital, Chengdu, China; ^2^Data Science R&D Center of Yanchang Technology, Chengdu, China

**Keywords:** endometrial cancer, lymph node metastasis, radiomics, machine learning, systematic review

## Abstract

**Background:**

The early identification of lymph node metastasis status in endometrial cancer (EC) is a serious challenge in clinical practice. Some investigators have introduced machine learning into the early identification of lymph node metastasis in EC patients. However, the predictive value of machine learning is controversial due to the diversity of models and modeling variables. To this end, we carried out this systematic review and meta-analysis to systematically discuss the value of machine learning for the early identification of lymph node metastasis in EC patients.

**Methods:**

A systematic search was conducted in Pubmed, Cochrane, Embase, and Web of Science until March 12, 2023. PROBAST was used to assess the risk of bias in the included studies. In the process of meta-analysis, subgroup analysis was performed according to modeling variables (clinical features, radiomic features, and radiomic features combined with clinical features) and different types of models in various variables.

**Results:**

This systematic review included 50 primary studies with a total of 103,752 EC patients, 12,579 of whom had positive lymph node metastasis. Meta-analysis showed that among the machine learning models constructed by the three categories of modeling variables, the best model was constructed by combining radiomic features with clinical features, with a pooled c-index of 0.907 (95%CI: 0.886-0.928) in the training set and 0.823 (95%CI: 0.757-0.890) in the validation set, and good sensitivity and specificity. The c-index of the machine learning model constructed based on clinical features alone was not inferior to that based on radiomic features only. In addition, logistic regression was found to be the main modeling method and has ideal predictive performance with different categories of modeling variables.

**Conclusion:**

Although the model based on radiomic features combined with clinical features has the best predictive efficiency, there is no recognized specification for the application of radiomics at present. In addition, the logistic regression constructed by clinical features shows good sensitivity and specificity. In this context, large-sample studies covering different races are warranted to develop predictive nomograms based on clinical features, which can be widely applied in clinical practice.

**Systematic review registration:**

https://www.crd.york.ac.uk/PROSPERO, identifier CRD42023420774.

## 1 Introduction

Endometrial cancer (EC) is the most common gynecological cancer in high-income countries. In 2020, 417,367 women were diagnosed with EC worldwide. Compared with low-income and middle-income countries, EC is more common in high-income regions. The regions with the highest EC diagnosis are North America and Western Europe, and the incidence rate of EC seems to be increasing rapidly (1, 2). EC is a serious threat to women’s lives. As of 2018, the incidence and mortality of women with EC in Europe were 19.2-20.2/100,000 and 2.0-3.7/100,000 (3, 4), respectively.

Surgery is the main treatment for patients with localized EC. However, whether lymphadenectomy is necessary during surgery is controversial, and whether para-aortic lymphadenectomy should be added to pelvic lymphadenectomy has been disputed (2, 5). Previously, all patients were advised to undergo complete standard lymphadenectomy (i.e., dissection and evaluation of pelvic and para-aortic lymph nodes), but this was associated with more side effects (6). Therefore, the effective preoperative assessment of lymph node metastasis is of profound significance in clinical practice. Unfortunately, there is a lack of efficient preoperative assessment methods. The Mayo criteria are widely applied in clinical practice for predicting the risk of lymph node metastasis in EC (7). However, its true prediction accuracy still needs to be further improved.

With the gradual improvement of statistical theory, researchers have gradually applied machine learning methods (especially supervised machine learning methods) into clinical practice, mainly for the diagnosis of disease status (8, 9), the prediction of disease occurrence (10, 11), or the prediction of prognosis (12, 13). In some fields, the accuracy of machine learning in screening or diagnosing diseases is not inferior to human clinical practice (14, 15). In this context, some investigators have tried to apply machine learning methods to the identification of preoperative lymph node metastasis in EC. However, machine learning includes diversified mathematical modeling methods (such as logistic regression, random forest, support vector machine, and artificial neural network), and machine learning models also involve a wide range of modeling variables (such as radiomic features, clinical features, and pathological imaging). As modeling methods and modeling variables are diversified, there is a lack of comprehensive and systematic understanding of the preoperative diagnostic value of machine learning for lymph node metastasis status in EC patients (16). Therefore, this systematic review and meta-analysis was conducted to explore the predictive value of machine learning for lymph node metastasis in EC patients. Also, we comprehensively summarized the effective predictive variables and compared the predictive values of clinical and radiomic features for lymph node metastasis in EC patients.

## 2 Methods

### 2.1 Study registration

This study followed the Preferred Reporting Items for Systematic Reviews and Meta-analyses (PRISMA 2020) and was prospectively registered on PROSPERO (ID: CRD42023420774).

### 2.2 Eligibility criteria

#### 2.2.1 Inclusion criteria

(1) Study subjects were patients diagnosed with EC;
(2) Study types were case-control study, cohort study, nested case-control study, and case-cohort study;
(3) Studies with a complete machine learning model for the prediction of lymph node metastasis status;
(4) Studies constructing a corresponding machine learning model but lacking external validation or independent validation set;
(5) Studies on different types of machine learning constructed from the same dataset;
(6) Studies reported in English.

#### 2.2.3 Exclusion criteria

(1) Study types were meta-analysis, review, guidelines, expert opinions, etc.;
(2) Studies with risk factors analyzed only but no complete risk model constructed;
(3) Studies lacking outcome indicators (Roc, c-statistic, c-index, sensitivity, specificity, accuracy, recovery, precision, confusion matrix, diagnostic fourfold table, F1 score, calibration curve) for the prediction accuracy of the risk model;
(4) Studies to validate maturity scale;
(5) Studies with the accuracy predicted by single factor.

### 2.3 Data sources and search strategy

A systematic search was conducted in Pubmed, Embase, Web of Science, and Cochrane until March 12, 2023, using subject terms and free terms. No restrictions were imposed on publication regions. The complete search strategy is shown in **Table S1**.

### 2.4 Study selection and data extraction

The retrieved studies were imported into Endnote X9 to automatically and manually remove duplicate publications. Then, the titles or abstracts were checked to obtain primary studies that were initially eligible. Finally, the full texts of the remaining studies were read to include primary studies that were eligible for this systematic overview.

Before data extraction, a standardized data extraction form was developed. Extracted data encompassed title, first author, publication year, study type (case-control, retrospective/prospective cohort study, nested cohort study, case-cohort study), patient source (single-center, multi-center, registry database), FIGO stage for tumor, number of cases with lymph node metastasis (training set, validation set), total number of cases, generation method of validation set (internal validation: random sampling, k-fold cross-validation, leave-one-out; external validation: prospective, multi-center; over-fitting method: k- fold cross-validation, bootstrap), missing value handling method, variable screening/feature selection method, types of mathematical models, and modeling variables.

Two investigators (RZL and YJY) independently screened the literature, extracted data, and cross-checked the data. In case of disagreement, a third investigator (LYQ) participated in discussions and decisions.

### 2.5 Risk of bias in the included studies

PROBAST was used to assess the risk of bias in the included studies, including several questions in four different domains: participants, predictive variables, results, and statistical analysis, which reflected the overall risk of bias and overall application (17). The four domains contained 2, 3, 6, and 9 questions in specificity respectively, each of which had three answers (yes/probably yes, no/probably no, and no available information). A domain was categorized as having a high risk of bias if at least one specific question in the domain indicated “no/probably no”. A domain was categorized as having a low risk of bias if all specific questions in the domain indicated “yes/probably yes”. A domain was categorized as having an unclear risk of bias if all specific questions in the domain indicated “no/probably no” with at least one “no available information”. The PROBAST was used to assess the machine learning models in the included literature.

Two investigators (RZL and YJY) independently assessed and cross-checked the risk of bias. In case of disagreement, a third investigator (LYQ) participated in discussions and decisions.

### 2.6 Outcomes

The outcome indicator in this systematic review was the c-index, which reflected the overall accuracy of the model. However, the c-index cannot reflect the accuracy of the model in predicting lymph node metastasis, especially when there is a serious imbalance in the number of lymph node metastasis and non-metastasis samples. Even if a high c-index is presented, it may be caused by the high accuracy of the model in predicting negative events (lymph node non-metastasis). Therefore, outcome indicators of this systematic review also included the sensitivity and specificity of machine learning models in predicting lymph node metastasis. In addition, we also summarized the modeling variables. As the machine learning models constructed clinically is mainly logistic regression, in order to try to construct the logistic regression risk equation for lymph node metastasis, outcome indicators also included the odds ratio (OR) of each modeling variable for constructing logistic regression.

### 2.7 Synthesis methods

The c-index, its standard error (SE), and 95% confidence interval (95%CI) should be provided for meta-analysis of c-index. However, since many included studies lacked the SE and 95%CI of c-index, the SE of c-index was estimated with reference to the study conducted by Debray TP et al. (18). This study also performed meta-analysis on sensitivity and specificity, for which a diagnostic fourfold table was required in the included studies. However, the included studies only provided outcome indicators such as sensitivity, specificity, precision and accuracy, so we developed the diagnostic fourfold table by combining the number of cases with lymph node metastasis and the total number of cases. In addition, some original studies only provided the receiver operating characteristic curve (Roc) of the machine learning model. In this case, we extracted the sensitivity and specificity on the Roc curve by using Origen2021, selected the sensitivity and specificity by using the best Youden’s index, and then developed the diagnostic fourfold table by combining the number of cases and the total number of cases. Moreover, the included studies converted continuous variables into categorical variables or remained them in the original continuous state when summarizing the OR values of modeling variables in Logistic regression, so we conducted meta-analysis of continuous variables.

In meta-analyses of c-index and OR values of modeling variables, a random-effects model was used when heterogeneity index I^2^≥50%, and a fixed-effects model was used when I^2^<50%. The meta-analysis of sensitivity and specificity was performed using a bivariate mixed-effects model.

In addition, the modeling variables consisted of clinical features, radiomic features, and radiomic + clinical features, and there were also diversified machine learning models. Therefore, subgroup analyses were conducted according to modeling variables and model types. This meta-analysis was performed in R4.2.0 (R development Core Team, Vienna, http://www.R-project.org), with a P value less than 0.05 indicating statistical significance.

## 3 Results

### 3.1 Study selection

**Figure 1:**
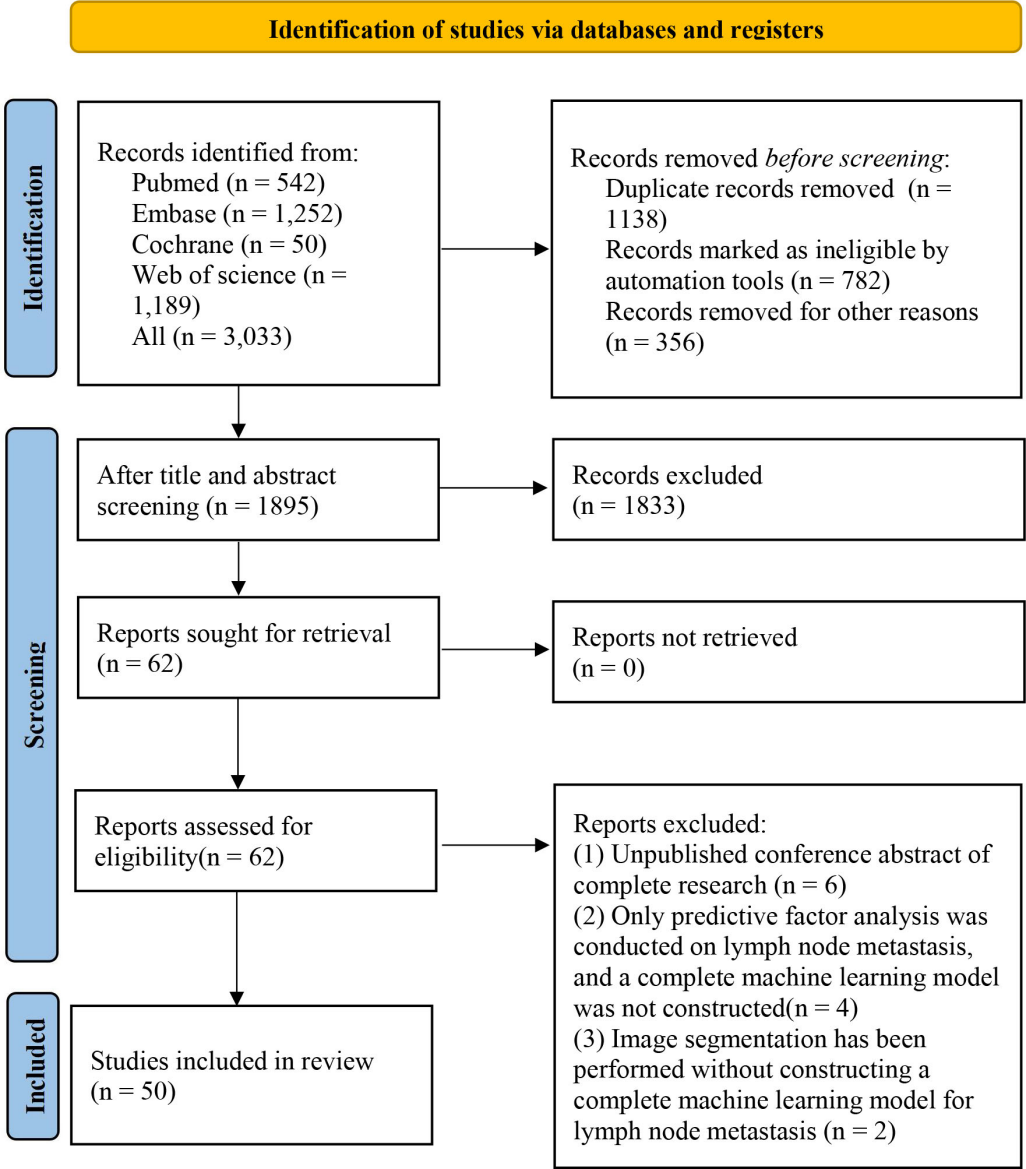
Literature screening process.

A total of 3,033 studies were retrieved, including 782 duplicate studies marked by Endnote. Endnote can only mark the literature with a completely consistent title and author’s writing style. However, a large number of duplicate studies had slight differences in these aspects, making it difficult to mark them automatically. Therefore, there were 356 studies that were manually identified duplicates. Then, after reading the titles or abstracts of the remaining literature, 62 primary studies were initially eligible, and their full texts were downloaded. After reading the full texts, 50 studies were finally included in this systematic review (19–68). The literature screening process is shown in **Figure 1**.

### 3.2 Study characteristics

The 50 included studies covered a total of 103,752 EC patients, of whom 12,579 EC patients had positive lymph node metastasis. The included studies were published from 2013 to 2023, mainly from 2016 to 2023. Study types included case-control study and cohort study. Among them, there were only 10 cohort studies (28, 35, 39, 49, 51–53, 62–64), 11 multicenter studies (26, 30, 33, 45, 46, 60–64, 66), and 1 study (40) from the SEER database, and the remaining studies were single-center studies. The median number of cases is 342 (IQR: 200~661), and the median number of cases in training sets is 300 (IQR: 154~533). Only 29 studies (19–21, 23, 25, 26, 29–33, 36–38, 40, 43, 45, 47–50, 52, 54, 55, 58, 60–63) had independent validation sets. Among them, 11 studies (21, 30, 31, 33, 36, 45, 55, 61–63, 65) adopted the method of external validation, and the remaining studies carried out internal validation by random sampling. Nine studies (20–22, 31, 32, 35, 45, 52) did not clearly describe the variable screening method and mainly adopted single-factor and multi-factor logistic regression.

14 studies (22, 23, 25, 26, 32, 34, 36, 37, 45, 47–50, 52, 55) had modeling variables from radiomic features and included a total of 27 models, which were divided into the following categories of diversified mathematical models: logistic regression (n=16), ridge regression (n=2), J48 (Decision tree (n=1)), random forest (n=1), support vector machine (n=2), XGBoost (n=1), artificial neural network (n=2), Hoeffding tree (n=1), and ResNet-18 (n=1). The remaining studies had modeling variables from clinical features and included a total of 41 models, which were divided into the following categories of diversified mathematical models: logistic regression (n=38), ridge regression (n=1), random forest (n=1), and support vector machine (n=1). The basic information is detailed in **Table 1**.

**Table 1:** Basic characteristics of the included literature.

No.	First author	Year of publication	Author country	Study type	Source of patients	Tumor staging	Cases with LNM	Total cases	Cases with LNM in the training set	Cases in the training set	The generation of the validation set	Cases with LNM in the validation set	Cases in the validation set	Model type
1	Yuzhen Huang	2023	China	Case control	Single center	FIGO:I-III	128	922	91	571	Random sampling	37	351	Logistic regression
2	Min Feng	2023	China	Case control	Single center	FIGO:I-IV	230	564	132	336	Random sampling	122	228	ResNet-18
3	Petra Vinklerová	2022	Czech	Case control	Single center	FIGO:I-IV	41	226			External validation	41	226	Bayesian networks
4	Çiğdem Soydal	2022	Turkey	Case control	Single center	FIGO:I-IV	30	157	30	157	10-fold cross-validation test			SVM Hoeffding Tree J48 ANN
5	Satoshi Otani	2022	Japan	Case control	Single center	FIGO:I-IV	30	200	23	150	Random sampling	7	50	XGBoost
6	Wen Lu	2022	China	Case control	Single center	FIGO:I-III	17	240	17	223				Logistic
7	Xuefei Liu	2022	China	Case control	Single center		42	704	20	350	Random sampling	22	354	Logistic
8	Xuefei Liu	2022	China	Case control	Multicenter		54	339	38	226	Random sampling	16	113	Logistic
9	Marcin Liro	2022	Poland	Case control	Single center	FIGO:I-III	46	114	46	114				Logistic
10	Yu-Yang Hsiao	2022	Taiwan-China	Cohort study	Single center	FIGO:I-IV	33	310	33	310				Logistic
11	Xingdan Guo	2022	China	Case control	Single center	FIGO:I-III	76	344	57	226	Random sampling	19	118	Logistic
12	Benedetta Guani	2022	Switzerland	Case control	Multicenter	FIGO:I-III	56	380	41	280	External validation 5-fold cross-validation test	15	100	Logistic
13	Marcel Grube	2022	Germany	Case control	Single center	FIGO:I-IV	20	247			External validation	20	247	Bayesian network
14	Juan Bo	2022	China	Case control	Single center	FIGO:I-IV	44	136	31	95	Random sampling	13	41	Logistic
15	Yuka Asami	2022	Japan	Case control	Multicenter		80	254	51	125	External validation	29	129	support vector machines random forests logistic regression
16	Kaiyue Zhang	2021	China	Case control	Single center	FIGO:I-IV	15	210	15	210	Cross validation			Logistic
17	Lan-Yan Yang	2021	China	Cohort study	Single center	FIGO:I-III	23	236						
18	Bi Cong Yan	2021	China	Case control	Single center	FIGO:I-IV	54	622	33	351	External validation	21	271	random forest
19	Yuquan Xu	2021	China	Case control	Single center	FIGO:I-IV	57	154	36	95	Random sampling	21	48	Logistic
20	Zhiling Wang	2021	China	Case control	Single center	FIGO:I-IV	105	1517	68	1000	Random sampling	37	517	Logistic
21	Marcin Liro	2021	Poland	Cohort study	Single center	FIGO:I-III	20	116	20	116				Logistic
22	Xingchen Li	2021	China	Case control	SEER	FIGO:I-IV	9,834	63836	4,917	42,558	Random sampling	2,404	21,278	Logistic
23	HuiFang Lei	2021	China	Case control	Single center		28	392	28	392				Logistic
24	Peng Jiang	2021	China	Case control	Single center	FIGO:I-II	83	651	83	651				Logistic
25	Peng Jiang	2021	China	Case control	Single center	FIGO:I-III	129	776	87	544	Random sampling	42	232	Logistic
26	Ying Zhang	2020	China	Case control	Single center	FIGO:I	46	507	46	507				Logistic
27	Casper Reijnen	2020	Netherlands	Case control	Multicenter	FIGO:I-IV	124	1593	53	763	External validation	52 19	446 384	Bayesian network
28	L S E Eriksson	2020	Sweden	Case control	Multicenter	FIGO:I-IV	127	691	127	691				Logistic
29	Cinzia Crivellaro	2020	Italy	Case control	Single center	FIGO:I-IV	35	167	8	60	Random sampling	29	107	Logistic
30	Jiaming Chen	2020	China	Case control	Single center	FIGO:I-II	20	150	15	104	Random sampling	5	46	Ridge regression
31	Hege F Berg	2020	Norway	Cohort study	Single center	FIGO:I-IV	33	299	27	243	Random sampling	6	56	Logistic
32	Xiaojuan Xu	2019	China	Case control	Single center	FIGO:I-IV	67	200	52	140	Random sampling	15	60	Logistic
33	Mehmet M Meydanli	2019	Turkey	Cohort study	Single center	FIGO:I-III	40	353	40	353				Logistic
34	Yangyang Kan	2019	China	Cohort study	Single center	FIGO:I-IV	58	143	44	100	Random sampling	14	43	SVM
35	Emre Günakan	2019	Turkey	Cohort study	Single center	FIGO:I-IV	102	762						LR
36	Yangyang Dong	2019	China	Case control	Single center		86	727	56	700		86	727	LR
37	Elisabetta De Bernardi	2018	Italy	Case control	Single center	FIGO:I-IV	25	115	16	86	External validation	9	29	ANN
38	Andressa M S Teixeira	2017	Brazil	Case control	Single center	FIGO:I-III	71	329	71	329				Logistic
39	Salih Taskın	2017	Turkey	Case control	Single center	FIGO:I-III	31	248	31	248				Logistic
40	Bingyi Yang	2016	China	Case control	Single center	FIGO:I-IV	65	570	39	300	Random sampling	26	200	Logistic
41	Erqi L Pollom	2016	Canada	Case control	Single center	FIGO:I-IV	13	296	13	296				Logistic
42	Martin Koskas (1)	2016	France	Case control	Multicenter	FIGO:I-IV	91	519				91	519	Logistic
43	Martin Koskas (2)	2016	France	Case control	Multicenter	FIGO:I-II		18793		18294	External validation		499	Logistic
44	Sofiane Bendifallah (1)	2015	France	Cohort study	Multicenter	FIGO:I-IV	124	1105	67	883	External validation	57	521	Logistic
45	Sofiane Bendifallah (2)	2015	France	Cohort study	Multicenter	FIGO:I-IV	65	523	65	523	External validation	18	140	Logistic
46	Martin Koskas	2014	France	Cohort study	Multicenter	FIGO:I-IV	14	167	14	167				Logistic
47	Sokbom Kang	2014	Korea	Case control	Single center	FIGO:I-IV	57	397	57	397	External validation			Logistic
48	Anna Luomaranta	2013	Finland	Case control	Multicenter	FIGO:I-IV	100	774	100	774				Logistic
49	M Koskas	2013	France	Case control	Single center		38	187	38	187				Logistic
50	Michelle F. Benoit	2020	US	Case control	Single center	FIGO:I-IV	52	490	52	490				Logistic

### 3.3 Risk of bias in the included studies

**Figure 2:**
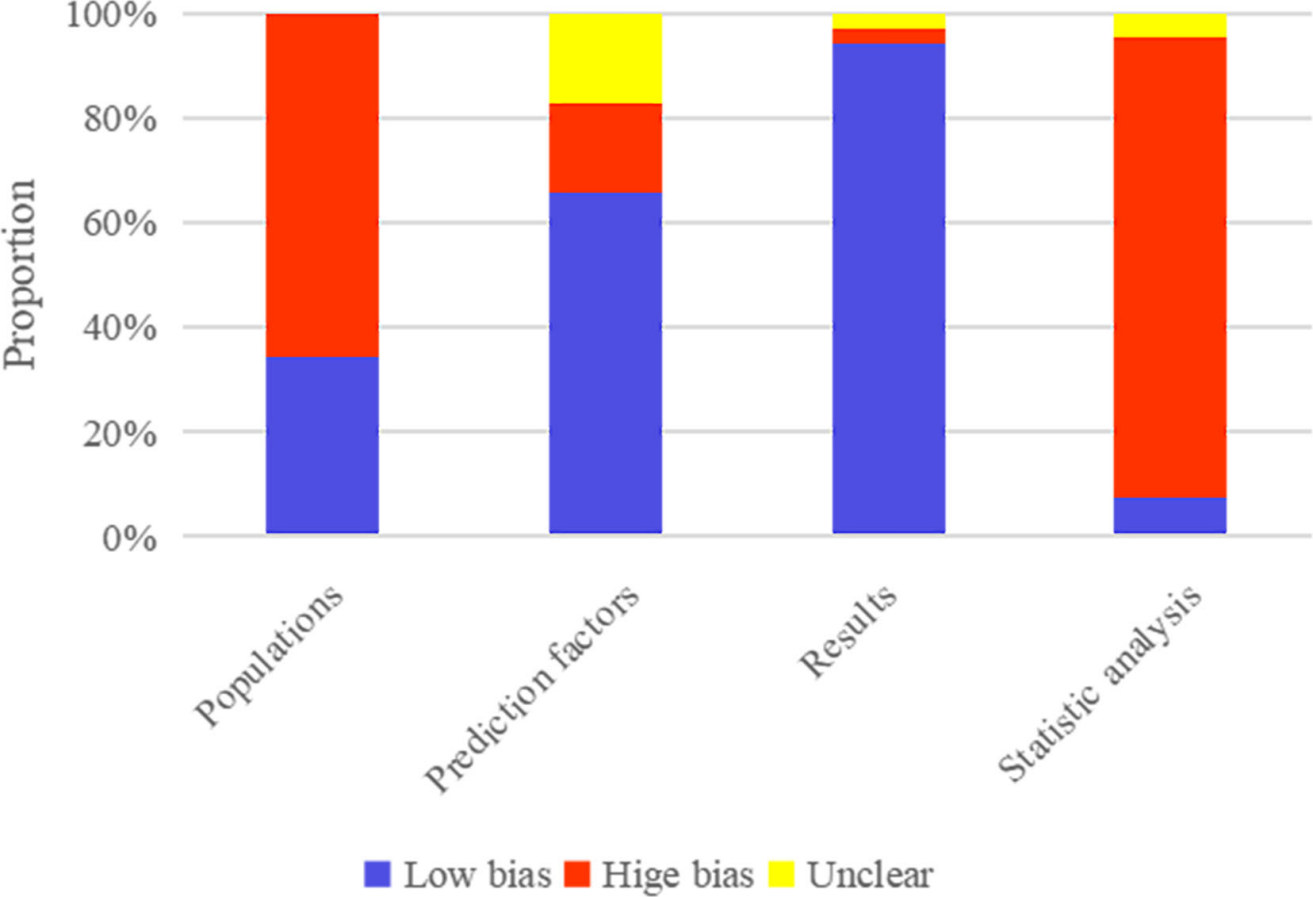
Results of risk of bias assessment of included machine-learning models by PROBAST.

The original studies included 39 case-control studies, based on which the machine learning model constructed was rated as high risk of bias for Populations by PROBAST. Also, it was unclear whether the assessment of predictive factors was carried out under the condition of known lymph node metastasis status in a large number of single-center case-control studies, based on which the machine learning model constructed was rated as high risk of bias for Prediction factors by PROBAST. In contrast, it was clear that the assessment of Results was carried out under the condition of confirmed lymph node metastasis status by biopsy in a large number of single-center case-control studies, based on which the machine learning model constructed was rated as high risk of bias for very few Results. In addition, according to Statistic analysis, the number of cases in the training set needed to meet EVP≥20. An independent validation set was required, and the number of cases in the validation set should be>100, leading to a main high risk of bias. The results of the risk of bias assessment are provided in **Figure 2**.

## 4 Meta-analysis

### 4.1 Mayo criteria

Eight datasets from the included studies were used to validate the accuracy of Meyo criteria for predicting lymph node metastasis in EC patients. The results of meta-analysis showed that the c-index in the training set was 0.690 (95%CI: 0.640-0.740), the sensitivity was 0.81 (95%CI: 0.66-0.90), and the specificity was 0.59 (95%CI: 0.38-0.77). The detailed results are shown in **Tables 2**, **3**.

**Table 2:** Meta-analysis results of c-index for predicting lymph node metastasis in EC patients using machine learning.

Variables	Model type	Training set		Validation set	
		n	c-index(95%CI)	n	c-index(95%CI)
Radiomics Features					
	Logistic regression	9	0.850(0.815~0.884)	6	0.818(0.775~0.861)
	Artificial neural network	2	0.762(0.687~0.837)	1	0.710(0.515~0.905)
	Support vector machine	2	0.727(0.661~0.793)	1	0.754(0.607~0.901)
	Ridge regression	1	0.730(0.605~0.855)		
	HoeffdingTree	1	0.688(0.585~0.791)		
	Convolutional neural network	1	0.701(0.599~0.803)		
	Overall	16	0.798(0.758~0.837)	8	0.810(0.770~0.850)
Radiomics + Clinical Features					
	Logistic regression	7	0.905(0.885~0.925)	4	0.842(0.793~0.891)
	Random forest	1	0.935(0.906~0.964)	2	0.903(0.866~0.939)
	XGBoost	1	0.800(0.680~0.920)	1	0.720(0.700~0.740)
	Ridge regression	1	0.800(0.700~0.900)	1	0.750(0.550~0.950)
	Convolutional neural network	1	0.938(0.913~0.963)	1	0.770(0.718~0.822)
	Overall	11	0.907(0.886~0.928)	9	0.823(0.757~0.890)
Clinical Features					
	Logistic regression	33	0.828(0.809~0.846)	14	0.806(0.758~0.853)
	Random forest	1	0.810(0.739~0.881)	1	0.820(0.735~0.905)
	Support vector machine	1	0.810(0.739~0.881)	1	0.760(0.663~0.857)
	Bayesian network			3	0.833(0.794~0.873)
	Ridge regression	1	0.610(0.458~0.762)		
	Overall	36	0.824(0.806~0.843)	19	0.793(0.756~0.829)
Mayo				8	0.690(0.640~0.740)

(1) n - Number of models; (2) Radiomics, radiomics+clinical features, and clinical features represent modeling variables, where radiomics+clinical features represent the combination of radiomics and clinical features as modeling variables.

**Table 3:** Meta-analysis results of sensitivity and specificity of machine learning in predicting lymph node metastasis in EC patients.

Variables	Model type	Training set			Validation set		
		n	Sen(95%CI)	Spe(95%CI)	n	Sen(95%CI)	Spe(95%CI)
Radiomics Features							
	Logistic regression	10	0.83(0.75~0.88)	0.79(0.76~0.82)	7	0.77(0.60~0.88)	0.86(0.75~0.93)
	Artificial neural network	2	0.77~0.86	0.66~0.94	1	0.89	0.75
	Support vector machine	2	0.75~0.81	0.75~0.87	1	0.71	0.72
	Ridge regression	1	0.7	0.86			
	HoeffdingTree	1	0.81	0.87			
	Convolutional neural network	2	0.80~0.83	0.90~0.91			
	Overall	18	0.82(0.79~0.85)	0.83(0.79~0.87)	9	0.77(0.64~0.87)	0.84(0.74~0.91)
Radiomics+Clinical Features							
	Logistic regression	7	0.90(0.84~0.94)	0.80(0.72~0.86)	6	0.78(0.62~0.88)	0.87(0.78~0.93)
	Artificial neural network	1	0.92	0.84	2	0.85~0.89	0.75~0.83
	Ridge regression	1	0.71	0.73			
	Convolutional neural network	1	0.83	0.91			
	Overall	10	0.88(0.84~0.92)	0.81(0.75~0.86)	6	0.81(0.70~0.89)	0.84(0.76~0.89)
Clinical Features							
	Logistic regression	31	0.81(0.78~0.84)	0.75(0.71~0.79)	13	0.74(0.66~0.80)	0.79(0.75~0.82)
	Random forest	1	0.67	0.78	1	0.48	0.87
	Bayesian network				2	0.87~0.94	0.68~0.70
	Overall	32	0.81(0.77~0.84)	0.75(0.71~0.79)	16	0.75(0.67~0.82)	0.78(0.74~0.82)
Mayo					7	0.81(0.66~0.90)	0.59(0.38~0.77)

### 4.2 Machine learning model based on clinical features alone for lymph node metastasis status

In the included studies, there were a total of 41 machine learning models constructed based on clinical features alone. The training set contained 36 machine learning models with a pooled c-index of 0.824 (95%CI: 0.806-0.843), and the validation set contained 19 machine learning models with a pooled c-index of 0.793 (95%CI: 0.756-0.829). The pooled sensitivity and specificity in the training set were 0.81 (95%CI: 0.77-0.84) and 0.75(95%CI: 0.71-0.79), and 0.75 (95%CI: 0.67-0.82) and 0.78 (95%CI: 0.74-0.82) in the validation set respectively. The detailed results are shown in **Tables 2**, **3**.

### 4.3 Machine learning model based on radiomic features alone for lymph node metastasis status

In the included studies, there were a total of 16 machine learning models constructed based on radiomic features alone. The pooled c-index was 0.798 (95%CI: 0.758-0.837) in the training set and 0.810 (95%CI: 0.770-0.850) in the validation set. The pooled sensitivity and specificity in the training set were 0.82 (95%CI: 0.79-0.85) and 0.83 (95%CI: 0.79-0.87), and 0.77 (95%CI: 0.64-0.87) and 0.84 (95%CI: 0.74-0.91) in the validation set, respectively. The detailed results are shown in **Tables 2**, **3**.

### 4.4 Machine learning model based on radiomic features combined with clinical features for lymph node metastasis status

In the included studies, there were a total of 11 machine learning models constructed based on radiomic features combined with clinical features. The pooled c-index was 0.907 (95%CI: 0.886-0.928) in the training set and 0.823 (95%CI: 0.757-0.890) in the validation set. The pooled sensitivity and specificity in the training set were 0.88 (95%CI: 0.84-0.92) and 0.83 (95%CI: 0.79-0.87), and 0.77 (95%CI: 0.64-0.87) and 0.84 (95%CI: 0.74-0.91) in the validation set, respectively. The detailed results are shown in **Tables 2**, **3**.

### 4.5 Subgroup analysis

Subgroup analyses were performed by the type of machine learning models constructed based on clinical features, radiomic features, and radiomic features combined with clinical features. The models for different modeling variables were mainly logistic regression, and most of the studies also constructed the visual Nomograms. The results of meta-analysis showed that logistic regression had a good predictive value not inferior to that of other machine learning models for the same modeling variable.

In the logistic regression constructed based on clinical features alone, the pooled c-index, sensitivity and specificity were 0.828 (95%CI: 0.809~0.846), 0.81 (95%CI: 0.78-0.84) and 0.75 (95% CI: 0.71-0.79) in the training set, and 0.806 (95%CI: 0.758-0.853), 0.81 (95%CI: 0.78-0.84) and 0.75 (95%CI: 0.71-0.79) in the validation set, respectively, as shown in **Figures S1** and **S2**.

In the logistic regression constructed based on radiomic features alone, the pooled c-index, sensitivity and specificity were 0.850 (95%CI: 0.815-0.884), 0.83 (95%CI: 0.75-0.88) and 0.79 (95%CI: 0.76-0.82) in the training set, and 0.818 (95%CI: 0.775-0.861), 0.77 (95%CI: 0.60-0.88) and 0.86 (95%CI: 0.75-0.93) in the validation set, respectively, as shown in **Figure S3** and **S4**.

In the logistic regression constructed based on radiomic features combined with clinical features, the pooled c-index, sensitivity and specificity in the training set were 0.905 (95%CI: 0.885-0.925), 0.90 (95%CI: 0.84-0.94) and 0.80 (95%CI: 0.72-0.86), respectively, and those in the validation set were 0.842 (95%CI: 0.793-0.891), 0.90 (95%CI: 0.84-0.94) and 0.80 (95%CI: 0.72-0.86), respectively, as shown in **Figure S5**, **S6**.

### 4.6 Modeling variables in logistic regression

Among the machine learning models constructed by the same type of modeling variables, logistic regression was not inferior to other models in the predictive value. We summarized the modeling variables included in logistic regression, and the results of meta-analysis showed that Grade, Histological type, Myometrial invasion, Cervical stromal invasion, LVSI, CA125, CA153, CA199, Ki67, P53, Tumor size, ER, Enlarged lymph nodes, Mitosis and SII were effective predictive variables (P<0.05) of lymph node metastasis status in EC, as shown in **Table 4** and **Figures S7**-**S12**.

**Table 4:** Meta-analysis results of the OR of modeling variables used to construct a Logistic regression model for predicting lymph node metastasis in EC.

Factors	Value	n	OR(95%CI)	I^2^(%)
Age				
	Per 1 year	2	1.026(0.941~1.120)	23.2
	>60	1	1.214(0.660~2.232)	NA
Grade				
	Grade2	7	2.258(2.039~2.500)	0.0
	Grade3	7	2.982(1.728~5.144)	81.0
	Grade2/3	5	1.983(1.164~3.379)	67.9
Histological type	Non-endometrioid	7	2.662(1.867~3.795)	65.5
Myometrial invasion	>50%	14	2.558(2.213~2.957)	19.7
Cervical stromal invasion	Yes	6	2.391(1.733~3.298)	10.0
LVSI(Lymphovascular invasion)	Positive	12	4.719(3.456~6.443)	59.4
CA125(Carbohydrate antigen 125)				
	>30	1	3.967(0.478~32.893)	NA
	>35	8	2.943(2.225~3.893)	10.0
	>40	2	8.129(4.448~14.853)	0.0
	>50	2	6.675(4.333~10.283)	0.0
CA153(Carbohydrate antigen 153)	>16.85	1	6.108(2.697~18.333)	NA
CA199(Carbohydrate antigen 199)	>18.88	1	3.765(1.505~9.418)	NA
Ki-67				
	per unit	3	1.028(1.014~1.041)	54.8
	>50%	3	2.397(1.385~4.151)	0.0
P53	Aberrant	4	2.402(1.143~5.049)	35.3
PR(Progesterone receptor)				
	Reduced by 1 unit	2	0.989(0.974~1.005)	79.6
	Negative	2	1.234(0.576~2.642)	0.0
Tumor size				
	Per 1 cm	3	1.348(1.128~1.611)	18.3
	>4cm	2	2.065(1.317~3.237)	0.0
	2-5cm	1	1.510(1.340~1.700)	NA
	5~10cm	1	2.710(2.390~3.060)	NA
	10cm~	1	3.380(2.900~3.950)	NA
ER(Estrogen receptor)				
	Negative	3	3.388(1.894~6.061)	0.0
	Reduced by 1 unit	2	0.979(0.962~0.995)	81.1
Enlarged lymph nodes	Positive	1	3.590(1.400~9.170)	NA
HGB(Hemoglobin)		1	0.983(0.967~0.999)	NA
MELF pattern	Present	1	1.977(0.508~7.695)	NA
Mitosis		1	3.202(1.650~6.214)	NA
SII(Systemic Immune-Inflammatory Index)	>636.74	1	3.996(1.808~8.833)	NA

## 5 Discussion

### 5.1 Clinical importance of preoperative assessment of lymph node metastasis

Preoperative identification of the status of lymph node metastases in EC patients is of profound clinical significance. For EC patients, some postoperative complications can seriously affect the quality of life of surviving patients. Among them, lymphoedema is one of the adverse complications that we need to pay attention to (69). Lymphadenectomy increases the risk of lymphoedema (70). Although the technique of sentinel lymph node (SLN) biopsy is used to infer the surgical staging of EC (71), however, researchers are still actively exploring some artificial intelligence-based lymph node metastasis detection tools.

### 5.2 Summary of the main findings

This study showed that the modeling variables for predicting lymph node metastasis status in EC patients included clinical features, radiomic features, and radiomic combined with clinical features. Among all types of modeling methods, logistic regression was mostly used to construct a nomogram, and it seems to have a c-index not inferior to that of other models in the training set and validation set. In addition, the c-index of the machine learning model constructed based on clinical features alone was not inferior to that of the machine learning model constructed based on radiomic features alone. In terms of the nomogram based on logistic regression, the c-index of the nomogram based on clinical features alone was close to that of the nomogram based on radiomic features alone. The machine learning method with the best predictive value was the one constructed based on radiomic features combined with clinical features, which was also applied to the nomogram.

### 5.3 Comparison with previous studies

Previous clinical research explored the accuracy of preoperative detection for lymph node metastasis in EC patients by using CT, MRI, PET/CT, ultrasound and other imaging approaches, mainly MRI and PET/CT. Bollineni VR et al. (72) systematically reviewed 13 original studies and reported that the sensitivity and specificity of 18F-FDG PET/CT in preoperative detection of lymph node metastasis in EC patients were 0.72 (95% CI: 0.55 ~ 0.98) and 0.92 (95% CI: 0.84 ~ 0.97), respectively. A recent study showed that the sensitivity of 18F-FDG PET and PET/CT in preoperative detection of lymph node metastasis in EC patients was 0.68 (95% CI: 0.63 ~ 0.73) and 0.96 (95% CI: 0.96 ~ 0.97), respectively (73). Qiu et al. (74) systematically reviewed 14 studies and found that the sensitivity and specificity of MRI for preoperative prediction of pelvic or/and para-aortic lymph node metastasis in EC patients were 0.59 (95%CI: 0.48 ~ 0.69) and 0.95 (95%CI: 0.93 ~ 0.96), while those of MRI for preoperative prediction of pelvic lymph node metastasis were 0.65 (95%CI: 0.51 ~ 0.77) and 0.95 (95%CI: 0.93 ~ 0.96). A systematic review by Luomaranta A et al. (75) on the preoperative detection of EC patients by MRI showed similar sensitivity and specificity to that reported by Qiu et al. The detection rate of lymph node metastasis in EC patients by ultrasound seems to be unsatisfactory (76). Thus, the preoperative detection of lymph node metastasis in EC patients by imaging approaches had a good specificity, but a seriously insufficient sensitivity. Our study showed that the machine learning method had a better sensitivity (> 0.8), and the machine learning model constructed based on clinical features had a higher sensitivity but a lower specificity to some extent.

In addition, this study showed that the Meyo criteria currently used in clinical practice had a high sensitivity, but its specificity was worrying. However, this finding was based on a small number of studies, and the identification value of Meyo criteria for lymph node metastasis in EC patients requires further verification.

Among diversified machine learning models, some had better prediction performance, such as convolutional neural network, support vector machine, and XGBoost (77, 78), but it seemed that the most popular machine learning model in clinical practice was still logistic regression. This is mainly because the nomogram can be constructed based on logistic regression. Nomogram features a simple application method and good performance in the visualization of results, which is very important for predicting lymph node metastasis in tumors, such as Briganti nomogram for prostate cancer (79). As shown in this study, logistic regression seemed to have a relatively good predictive value. Therefore, follow-up studies can try to develop more general nomograms for predicting lymph node metastasis in EC patients.

Modeling variables are of critical importance for improving the accuracy of machine learning. However, only a few studies summarized the evidence in this regard. The systematic review by Reijnen C et al. (80) showed that CA-125 and thrombocytosis were associated with the risk of lymph node metastasis in EC patients, and the systematic review by Fu et al. (81) reported that tumor diameter was also related to lymph node metastasis. Therefore, the lack of comprehensive independent predictors for lymph node metastasis in EC patients has posed a challenge to the early identification of lymph node metastasis status in EC patients. In this study, we summarized the modeling variables included in machine learning. Since the risk model constructed based on clinical features alone also had good sensitivity (>0.8), risk equations or predictive nomograms for preoperative prediction of lymph node metastasis in EC patients can be constructed based on this study.

The FIGO 2023 staging system (82) classifies lymph node metastases to micrometastasis and macrometastasis, in which IIIC1 was metastasis to the pelvic lymph nodes (IIIC1i: micrometastasis, IIIC1ii: macrometastasis), IIIC2 was metastasis to para-aortic lymph nodes up to the renal vessels, with or without metastasis to the pelvic lymph nodes (IIIC2i: micrometastasis, IIIC2ii: macrometastasis). SLN biopsy is an appropriate alternative to systematic lymphadenectomy, and ultrastaging provides more sensitive and accurate identification of lymphatic disease than standard lymph node dissection. SLN biopsy may also be considered for low/low intermediate-risk patients to rule out occult lymph node metastases and to identify disease that is truly confined to the uterus. Therefore, the ESGO-ESTRO-ESP guidelines allow a SLN approach for all EC patients, which is recognized by FIGO. Although, the value of machine learning for the identification of lymph node metastatic status in EC patients was systematically described in our study, the detection of lymph node metastatic site and extent is also necessary. Future studies could explore the identification of metastatic status for SLN.

### 5.4 Strengths and limitations of the study

The strengths of this study lie in that it was the first systematic review on the preoperative diagnostic value of machine learning for lymph node metastasis in EC patients, and it summarized the existing main modeling variables (clinical features, radiomic features), so as to provide guidance and references for the development of clinical risk tools in the future. However, there are still some limitations in this study. Firstly, most of the included studies focused on logistic regression, with less exploration on other models, making it difficult to summarize their applied value. Secondly, in the included studies, the validation method of the models was mainly internal validation with random sampling, which likely restricted the promotion of the model to other fields. Especially for models based on radiomic features, it poses a serious challenge, since the radiomic features are seriously affected by the experience of radiologists, and the configuration of radiation devices. Thirdly, the included studies were mainly case-control studies, some of which had a small sample size, raising a concern about the stability of the model. Fourthly, The Cancer Genome Atlas (TCGA) classifies EC into four distinct molecular categories: POLE ultramutated (POLEmut), high microsatellite instability (MSI-H) or mismatch repair defective (MSI-H or MMRd), copy number low or no specific molecular profiling (CNL or NSMP), and copy number high or p53 abnormal (CNH or p53abn). However, the original studies included did not strictly differentiate between molecular subtypes (POLEmut, MMRd, NSMP, and p53bn), which resulted in our systematic review failing to provide corresponding evidence. Finally, we only included studies that constructed machine learning for detecting lymph node metastasis and aggregated interpretable clinical features and associations with lymph node metastasis. However, we did not include studies that only analyzed risk factors. Thus, the pooled results may have missed a small number of other clinical features.

## 6 Conclusions

The machine learning model is feasible for preoperative prediction of the lymph node metastasis status of EC patients, and the visual nomogram of logistic regression constructed based on clinical features has favorable sensitivity and specificity. In addition, models based on radiomic features combined with clinical features have a better predictive value. Large-sample studies covering different races are warranted to develop predictive nomograms based on clinical features, which can be widely applied in clinical practice. In view of the excellent predictive performance of machine learning models constructed based on radiomic features combined with clinical features, we also look forward to accelerating the development and application of radiomic features and proposing standardized criteria for their application, so as to develop intelligent diagnosis of complex disease status and intelligent prediction of disease prognosis based on radiomic features.

## Data availability statement

The original contributions presented in the study are included in the article/**Supplementary Material**. Further inquiries can be directed to the corresponding authors.

## Author contributions

ZR: Conceptualization, Data curation, Formal analysis, Methodology, Resources, Writing – original draft, Writing – review & editing. BC: Conceptualization, Investigation, Supervision, Validation, Writing – original draft. CH: Formal analysis, Investigation, Supervision, Validation, Writing – original draft. JYY: Data curation, Formal analysis, Investigation, Resources, Writing – original draft. JD: Software, Supervision, Validation, Visualization, Writing – original draft. YC: Formal analysis, Investigation, Validation, Visualization, Writing – original draft. JLY: Data curation, Formal analysis, Investigation, Validation, Writing – original draft. YL: Formal analysis, Methodology, Software, Visualization, Writing – review & editing.
