# Supplementary material for: The value of machine learning in preoperative identification of lymph node metastasis status in endometrial cancer: a systematic review and meta-analysis
Source: Front Oncol. 2023 Dec 20;13:1289050. doi: 10.3389/fonc.2023.1289050 (PMC10761539; doi:10.3389/fonc.2023.1289050)
Supplement: Supplementary file 1 [file DataSheet_1.docx]

**Supplementary materials**

# Table S1 Literature search strategy

**1.Pubmed**

| Search number | Query | Results |
| --- | --- | --- |
| #1 | "Endometrial Neoplasms"[Mesh] | 25,427 |
| #2 | ((((((((((((((((((((((((((Endometrial Neoplasms[Title/Abstract]) OR (Endometrial Neoplasm[Title/Abstract])) OR (Endometrial Carcinoma[Title/Abstract])) OR (Endometrial Carcinomas[Title/Abstract])) OR (Endometrial Cancer[Title/Abstract])) OR (Endometrial Cancers[Title/Abstract])) OR (Endometrium Cancer[Title/Abstract])) OR (Cancer of the Endometrium[Title/Abstract])) OR (Cancer of the Endometrium[Title/Abstract])) OR (Endometrium Carcinoma[Title/Abstract])) OR (Endometrium Carcinomas[Title/Abstract])) OR (Cancer of Endometrium[Title/Abstract])) OR (Endometrium Cancers[Title/Abstract])) OR (endometrium tumor[Title/Abstract])) OR (endometrial tumor[Title/Abstract])) OR (endometrial tumour[Title/Abstract])) OR (endometrioma[Title/Abstract])) OR (endometrium tumour[Title/Abstract])) OR (Endometrioid Carcinoma[Title/Abstract])) OR (Endometrioid Carcinomas[Title/Abstract])) OR (Endometrioid Adenocarcinomas[Title/Abstract])) OR (Endometrioid Adenocarcinoma[Title/Abstract])) OR (Carcinomas, Endometrioid[Title/Abstract])) OR (Endometrioid Carcinoma[Title/Abstract])) OR (Endometrioid Carcinomas[Title/Abstract])) OR (Endometrioid Adenocarcinomas[Title/Abstract])) OR (Endometrioid Adenocarcinoma[Title/Abstract]) | 34,776 |
| #3 | ("Endometrial Neoplasms"[Mesh]) OR (((((((((((((((((((((((((((Endometrial Neoplasms[Title/Abstract]) OR (Endometrial Neoplasm[Title/Abstract])) OR (Endometrial Carcinoma[Title/Abstract])) OR (Endometrial Carcinomas[Title/Abstract])) OR (Endometrial Cancer[Title/Abstract])) OR (Endometrial Cancers[Title/Abstract])) OR (Endometrium Cancer[Title/Abstract])) OR (Cancer of the Endometrium[Title/Abstract])) OR (Cancer of the Endometrium[Title/Abstract])) OR (Endometrium Carcinoma[Title/Abstract])) OR (Endometrium Carcinomas[Title/Abstract])) OR (Cancer of Endometrium[Title/Abstract])) OR (Endometrium Cancers[Title/Abstract])) OR (endometrium tumor[Title/Abstract])) OR (endometrial tumor[Title/Abstract])) OR (endometrial tumour[Title/Abstract])) OR (endometrioma[Title/Abstract])) OR (endometrium tumour[Title/Abstract])) OR (Endometrioid Carcinoma[Title/Abstract])) OR (Endometrioid Carcinomas[Title/Abstract])) OR (Endometrioid Adenocarcinomas[Title/Abstract])) OR (Endometrioid Adenocarcinoma[Title/Abstract])) OR (Carcinomas, Endometrioid[Title/Abstract])) OR (Endometrioid Carcinoma[Title/Abstract])) OR (Endometrioid Carcinomas[Title/Abstract])) OR (Endometrioid Adenocarcinomas[Title/Abstract])) OR (Endometrioid Adenocarcinoma[Title/Abstract])) | 41,267 |
| #4 | "Machine Learning"[Mesh] | 54,673 |
| #5 | ((((((((((((((((((((((((((machine learning[Title/Abstract]) OR (Transfer Learning[Title/Abstract])) OR (Deep learning[Title/Abstract])) OR (Ensemble Learning[Title/Abstract])) OR (artificial intelligence[Title/Abstract])) OR (Prediction model[Title/Abstract])) OR (Prediction models[Title/Abstract])) OR (random forest[Title/Abstract])) OR (neural network[Title/Abstract])) OR (neural networks[Title/Abstract])) OR (CNN[Title/Abstract])) OR (Support vector machine[Title/Abstract])) OR (SVM[Title/Abstract])) OR (Gradient Boosting Machine[Title/Abstract])) OR (Nomogram[Title/Abstract])) OR (XGBoost[Title/Abstract])) OR (Adaboost[Title/Abstract])) OR (Perceptrons[Title/Abstract])) OR (Perceptron[Title/Abstract])) OR (Decision tree[Title/Abstract])) OR (ResNet-50[Title/Abstract])) OR (ResNet[Title/Abstract])) OR (Radiomics[Title/Abstract])) OR (Radiomic[Title/Abstract])) OR (Naive Bayesian[Title/Abstract])) OR (Risk Prediction[Title/Abstract])) OR (Risk-Prediction[Title/Abstract]) | 274,801 |
| #6 | ("Machine Learning"[Mesh]) OR (((((((((((((((((((((((((((machine learning[Title/Abstract]) OR (Transfer Learning[Title/Abstract])) OR (Deep learning[Title/Abstract])) OR (Ensemble Learning[Title/Abstract])) OR (artificial intelligence[Title/Abstract])) OR (Prediction model[Title/Abstract])) OR (Prediction models[Title/Abstract])) OR (random forest[Title/Abstract])) OR (neural network[Title/Abstract])) OR (neural networks[Title/Abstract])) OR (CNN[Title/Abstract])) OR (Support vector machine[Title/Abstract])) OR (SVM[Title/Abstract])) OR (Gradient Boosting Machine[Title/Abstract])) OR (Nomogram[Title/Abstract])) OR (XGBoost[Title/Abstract])) OR (Adaboost[Title/Abstract])) OR (Perceptrons[Title/Abstract])) OR (Perceptron[Title/Abstract])) OR (Decision tree[Title/Abstract])) OR (ResNet-50[Title/Abstract])) OR (ResNet[Title/Abstract])) OR (Radiomics[Title/Abstract])) OR (Radiomic[Title/Abstract])) OR (Naive Bayesian[Title/Abstract])) OR (Risk Prediction[Title/Abstract])) OR (Risk-Prediction[Title/Abstract])) | 279,758 |
| #7 | (("Endometrial Neoplasms"[Mesh]) OR (((((((((((((((((((((((((((Endometrial Neoplasms[Title/Abstract]) OR (Endometrial Neoplasm[Title/Abstract])) OR (Endometrial Carcinoma[Title/Abstract])) OR (Endometrial Carcinomas[Title/Abstract])) OR (Endometrial Cancer[Title/Abstract])) OR (Endometrial Cancers[Title/Abstract])) OR (Endometrium Cancer[Title/Abstract])) OR (Cancer of the Endometrium[Title/Abstract])) OR (Cancer of the Endometrium[Title/Abstract])) OR (Endometrium Carcinoma[Title/Abstract])) OR (Endometrium Carcinomas[Title/Abstract])) OR (Cancer of Endometrium[Title/Abstract])) OR (Endometrium Cancers[Title/Abstract])) OR (endometrium tumor[Title/Abstract])) OR (endometrial tumor[Title/Abstract])) OR (endometrial tumour[Title/Abstract])) OR (endometrioma[Title/Abstract])) OR (endometrium tumour[Title/Abstract])) OR (Endometrioid Carcinoma[Title/Abstract])) OR (Endometrioid Carcinomas[Title/Abstract])) OR (Endometrioid Adenocarcinomas[Title/Abstract])) OR (Endometrioid Adenocarcinoma[Title/Abstract])) OR (Carcinomas, Endometrioid[Title/Abstract])) OR (Endometrioid Carcinoma[Title/Abstract])) OR (Endometrioid Carcinomas[Title/Abstract])) OR (Endometrioid Adenocarcinomas[Title/Abstract])) OR (Endometrioid Adenocarcinoma[Title/Abstract]))) AND (("Machine Learning"[Mesh]) OR (((((((((((((((((((((((((((machine learning[Title/Abstract]) OR (Transfer Learning[Title/Abstract])) OR (Deep learning[Title/Abstract])) OR (Ensemble Learning[Title/Abstract])) OR (artificial intelligence[Title/Abstract])) OR (Prediction model[Title/Abstract])) OR (Prediction models[Title/Abstract])) OR (random forest[Title/Abstract])) OR (neural network[Title/Abstract])) OR (neural networks[Title/Abstract])) OR (CNN[Title/Abstract])) OR (Support vector machine[Title/Abstract])) OR (SVM[Title/Abstract])) OR (Gradient Boosting Machine[Title/Abstract])) OR (Nomogram[Title/Abstract])) OR (XGBoost[Title/Abstract])) OR (Adaboost[Title/Abstract])) OR (Perceptrons[Title/Abstract])) OR (Perceptron[Title/Abstract])) OR (Decision tree[Title/Abstract])) OR (ResNet-50[Title/Abstract])) OR (ResNet[Title/Abstract])) OR (Radiomics[Title/Abstract])) OR (Radiomic[Title/Abstract])) OR (Naive Bayesian[Title/Abstract])) OR (Risk Prediction[Title/Abstract])) OR (Risk-Prediction[Title/Abstract]))) | 542 |

**2.Cochrane**

| Search number | Query | Results |
| --- | --- | --- |
| #1 | MeSH descriptor: [Endometrial Neoplasms] explode all trees | 996 |
| #2 | (Endometrial Neoplasms):ti,ab,kw OR (Endometrial Neoplasm):ti,ab,kw OR (Endometrial Carcinoma):ti,ab,kw OR (Endometrial Carcinomas):ti,ab,kw OR (Endometrial Cancer):ti,ab,kw | 2,836 |
| #3 | (Endometrial Cancers):ti,ab,kw OR (Endometrium Cancer):ti,ab,kw OR (Cancer of the Endometrium):ti,ab,kw OR (Carcinoma of Endometrium):ti,ab,kw OR (Endometrium Carcinoma):ti,ab,kw | 1,673 |
| #4 | (Endometrium Carcinomas):ti,ab,kw OR (Endometrium Cancers):ti,ab,kw OR (Cancer of Endometrium):ti,ab,kw OR (endometrium tumor):ti,ab,kw OR (endometrial tumor):ti,ab,kw | 1,883 |
| #5 | (endometrial tumour):ti,ab,kw OR (endometrioma):ti,ab,kw OR (endometrium tumour):ti,ab,kw OR (Endometrioid Carcinoma):ti,ab,kw OR (Endometrioid Carcinomas):ti,ab,kw | 1,342 |
| #6 | (Endometrioid Adenocarcinomas):ti,ab,kw OR (Endometrioid Adenocarcinoma):ti,ab,kw OR (Endometrioid Carcinoma):ti,ab,kw OR (Endometrioid Carcinomas):ti,ab,kw OR (Endometrioid Adenocarcinomas):ti,ab,kw | 360 |
| #7 | (Endometrioid Adenocarcinoma):ti,ab,kw | 160 |
| #8 | #1 OR #2 OR #3 OR #4 OR #5 OR #6 OR #7 | 3,739 |
| #9 | MeSH descriptor: [Machine Learning] explode all trees | 712 |
| #10 | (machine learning):ti,ab,kw OR (Transfer Learning):ti,ab,kw OR (Deep learning):ti,ab,kw OR (Ensemble Learning):ti,ab,kw OR (artificial intelligence):ti,ab,kw | 5,810 |
| #11 | (Prediction model):ti,ab,kw OR (Prediction models):ti,ab,kw OR (random forest):ti,ab,kw OR (neural network):ti,ab,kw OR (neural networks):ti,ab,kw | 9,891 |
| #12 | (CNN):ti,ab,kw OR (Support vector machine):ti,ab,kw OR (SVM):ti,ab,kw OR (Gradient Boosting Machine):ti,ab,kw OR (Nomogram):ti,ab,kw | 2,211 |
| #13 | (XGBoost):ti,ab,kw OR (Adaboost):ti,ab,kw OR (Perceptrons):ti,ab,kw OR (Perceptron):ti,ab,kw OR (Decision tree):ti,ab,kw | 1,000 |
| #14 | (ResNet-50):ti,ab,kw OR (ResNet):ti,ab,kw OR (Radiomics):ti,ab,kw OR (Radiomic):ti,ab,kw OR (Naive Bayesian):ti,ab,kw | 669 |
| #15 | (Risk Prediction):ti,ab,kw OR (Risk-Prediction):ti,ab,kw | 6,093 |
| #16 | #9 OR #10 OR #11 OR #12 OR #13 OR #14 OR #15 | 18,545 |
| #17 | #8 AND #16 | 50 |

**3.Embase**

| Search number | Query | Results |
| --- | --- | --- |
| #1 | 'endometrium tumor'/exp | 83,065 |
| #2 | 'endometrial neoplasms':ab,ti OR 'endometrial neoplasm':ab,ti OR 'endometrial carcinoma':ab,ti OR 'endometrial carcinomas':ab,ti OR 'endometrial cancer':ab,ti OR 'endometrial cancers':ab,ti OR 'endometrium cancer':ab,ti OR 'cancer of the endometrium':ab,ti OR 'carcinoma of endometrium':ab,ti OR 'endometrium carcinoma':ab,ti OR 'endometrium carcinomas':ab,ti OR 'cancer of endometrium':ab,ti OR 'endometrium cancers':ab,ti OR 'endometrium tumor':ab,ti OR 'endometrial tumor':ab,ti OR 'endometrial tumour':ab,ti OR endometrioma:ab,ti OR 'endometrium tumour':ab,ti OR 'endometrioid carcinoma':ab,ti OR 'endometrioid carcinomas':ab,ti OR 'endometrioid adenocarcinomas':ab,ti OR 'endometrioid adenocarcinoma':ab,ti | 51,148 |
| #3 | #1 OR #2 | 89,331 |
| #4 | 'machine learning'/exp | 371,024 |
| #5 | 'machine learning':ab,ti OR 'transfer learning':ab,ti OR 'deep learning':ab,ti OR 'ensemble learning':ab,ti OR 'artificial intelligence':ab,ti OR 'prediction model':ab,ti OR 'prediction models':ab,ti OR 'random forest':ab,ti OR 'neural network':ab,ti OR 'neural networks':ab,ti OR cnn:ab,ti OR 'support vector machine':ab,ti OR svm:ab,ti OR 'gradient boosting machine':ab,ti OR nomogram:ab,ti OR xgboost:ab,ti OR adaboost:ab,ti OR perceptrons:ab,ti OR perceptron:ab,ti OR 'decision tree':ab,ti OR 'resnet 50':ab,ti OR resnet:ab,ti OR radiomics:ab,ti OR radiomic:ab,ti OR 'naive bayesian':ab,ti OR 'risk prediction':ab,ti | 323,933 |
| #6 | #4 OR #5 | 518,862 |
| #7 | #3 AND #6 | 1,252 |

**4.Web of science**

| Search number | Query | Results |
| --- | --- | --- |
| #1 | Endometrial Neoplasms (Topic) OR Endometrial Neoplasm (Topic) OR Endometrial Carcinoma (Topic) OR Endometrial Carcinomas (Topic) OR Endometrial Cancer (Topic) OR Endometrial Cancers (Topic) OR Endometrium Cancer (Topic) OR Cancer of the Endometrium (Topic) OR Carcinoma of Endometrium (Topic) OR Endometrium Carcinoma (Topic) OR Endometrium Carcinomas (Topic) OR Cancer of Endometrium (Topic) OR Endometrium Cancers (Topic) OR endometrium tumor (Topic) OR endometrial tumor (Topic) OR endometrial tumour (Topic) OR endometrioma (Topic) OR endometrium tumour (Topic) OR Endometrioid Carcinoma (Topic) OR Endometrioid Carcinomas (Topic) OR Endometrioid Adenocarcinomas (Topic) OR Endometrioid Adenocarcinoma (Topic) OR Endometrioid Carcinoma (Topic) OR Endometrioid Carcinomas (Topic) OR Endometrioid Adenocarcinomas (Topic) OR Endometrioid Adenocarcinoma (Topic) | 58,381 |
| #2 | machine learning (Topic) OR Transfer Learning (Topic) OR Deep learning (Topic) OR Ensemble Learning (Topic) OR artificial intelligence (Topic) OR Prediction model (Topic) OR Prediction models (Topic) OR random forest (Topic) OR neural network (Topic) OR neural networks (Topic) OR CNN (Topic) OR Support vector machine (Topic) OR SVM (Topic) OR Gradient Boosting Machine (Topic) OR Nomogram (Topic) OR XGBoost (Topic) OR Adaboost (Topic) OR Perceptrons (Topic) OR Perceptron (Topic) OR Decision tree (Topic) OR ResNet-50 (Topic) OR ResNet (Topic) OR Radiomics (Topic) OR Radiomic (Topic) OR Naive Bayesian (Topic) OR Risk Prediction (Topic) OR Risk-Prediction (Topic) | 2,070,485 |
| #3 | #1 AND #2 | 1,189 |


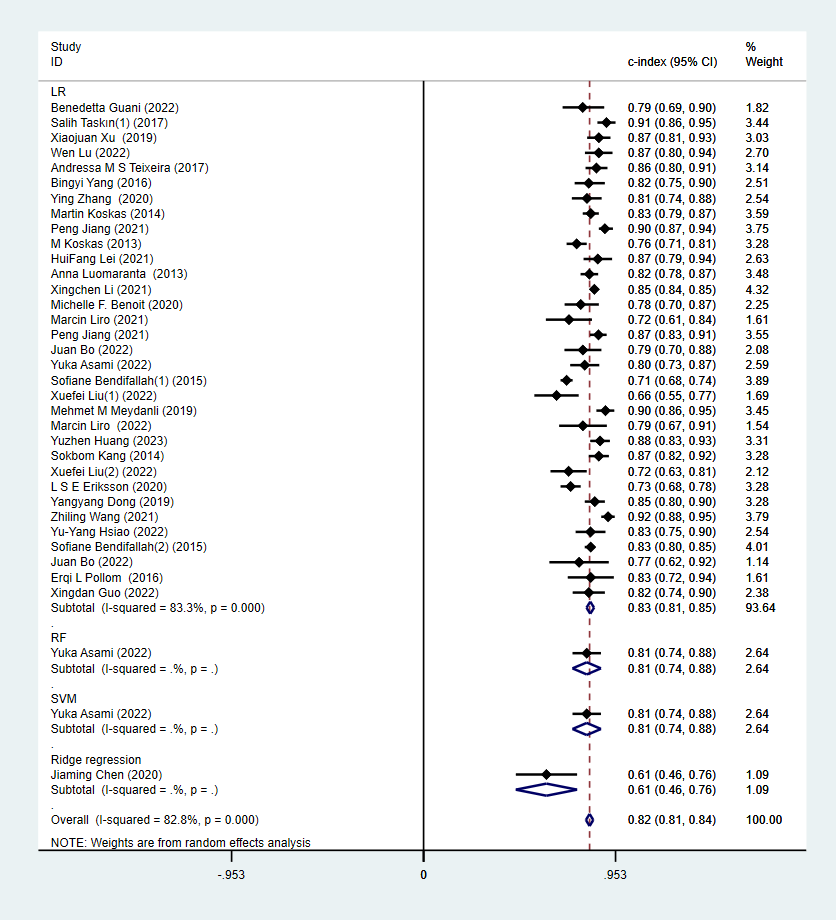


FigureS1 Forest plot of meta-analysis of machine learning models for endometrial carcinoma with lymph node metastasis constructed based on clinical features in the training set


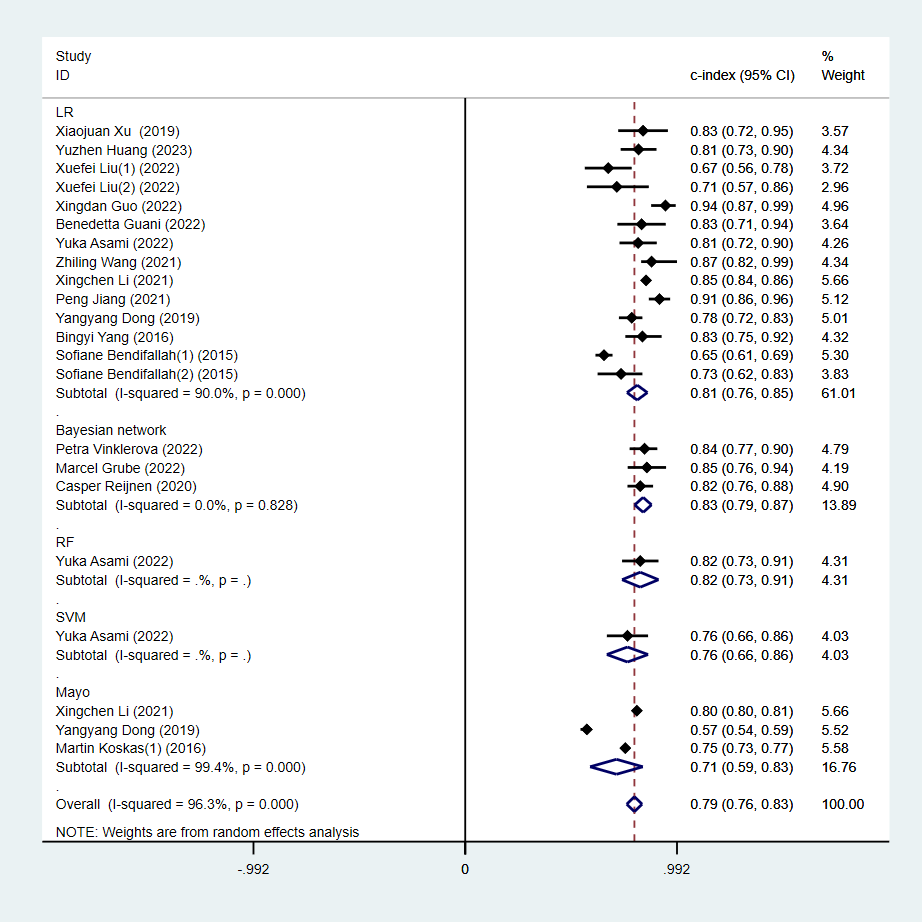


FigureS2 Forest plot of meta-analysis of machine learning models for endometrial carcinoma with lymph node metastasis constructed based on clinical features in the validation set


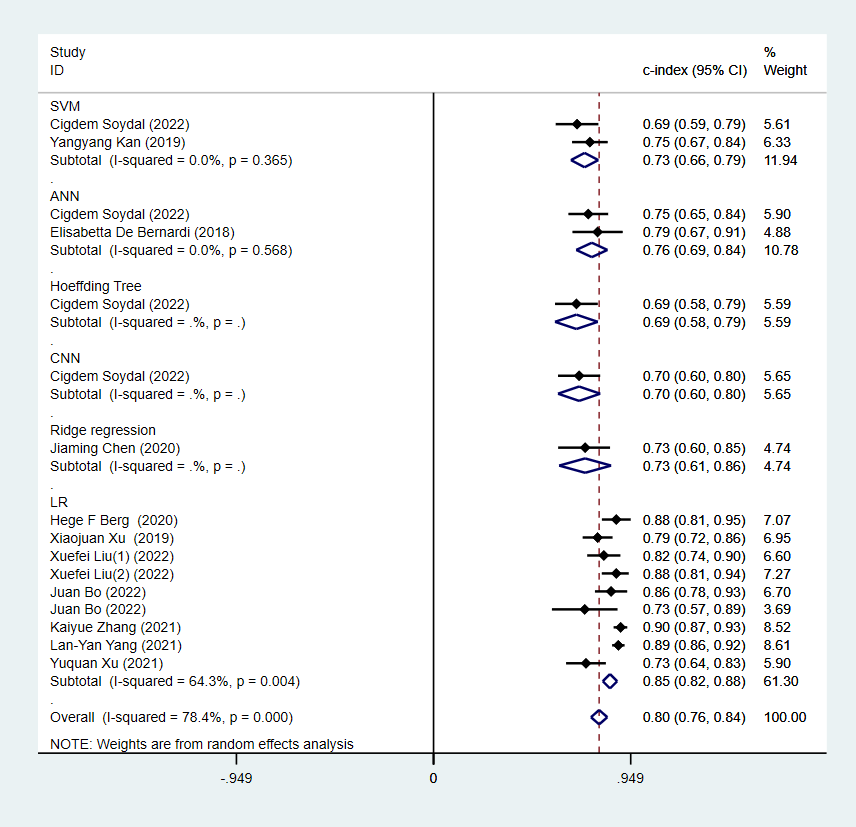


FigureS3 Forest plot of meta-analysis of machine learning models for endometrial carcinoma with lymph node metastasis constructed based on radiomic features in the training set


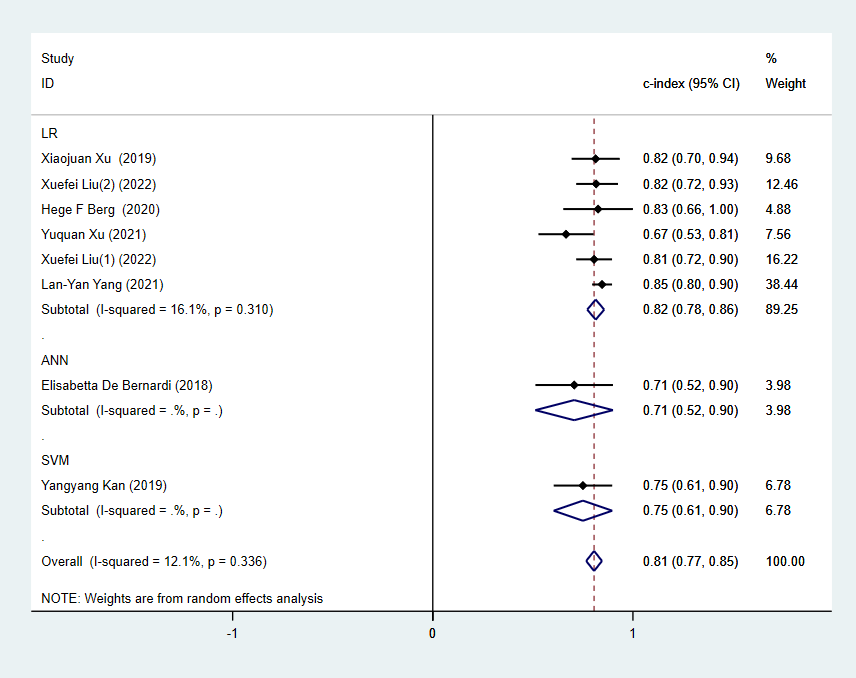


FigureS4 Forest plot of meta-analysis of machine learning models for endometrial carcinoma with lymph node metastasis constructed based on radiomic features in the validation set


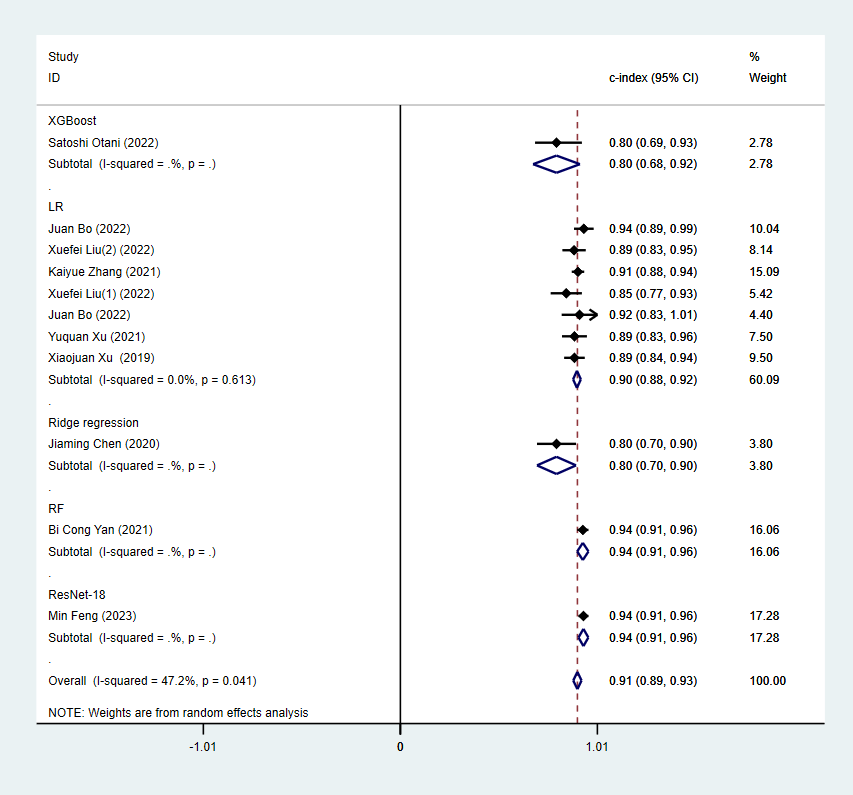


FigureS5 Forest plot of meta-analysis of machine learning models for endometrial carcinoma with lymph node metastasis constructed based on both clinical features and radiomic features in the training set


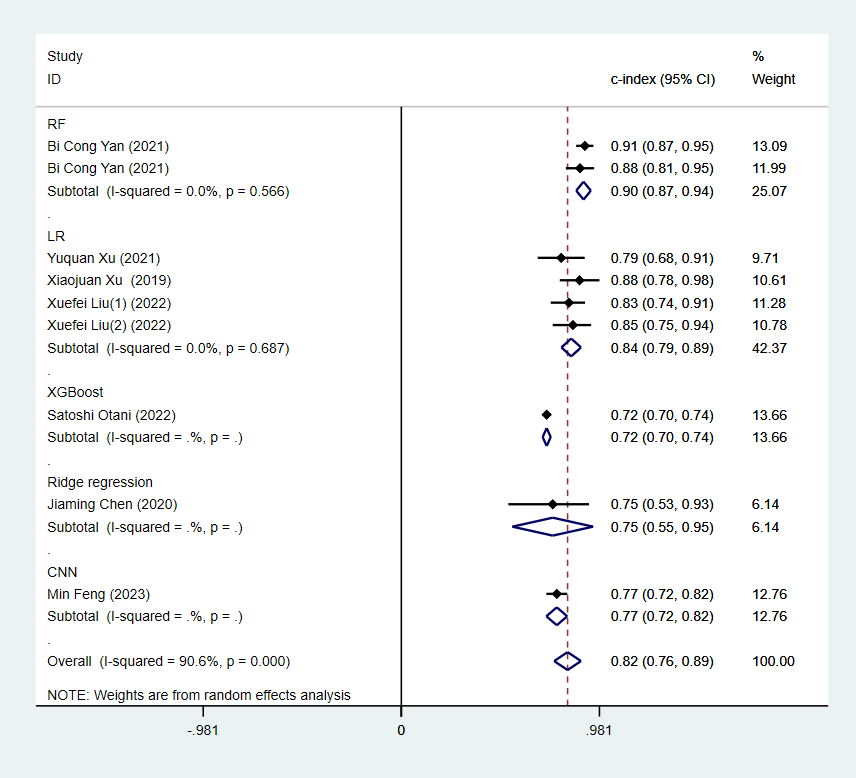


FigureS6 Forest plot of meta-analysis of machine learning models for endometrial carcinoma with lymph node metastasis constructed based on both clinical features and radiomic features in the validation set


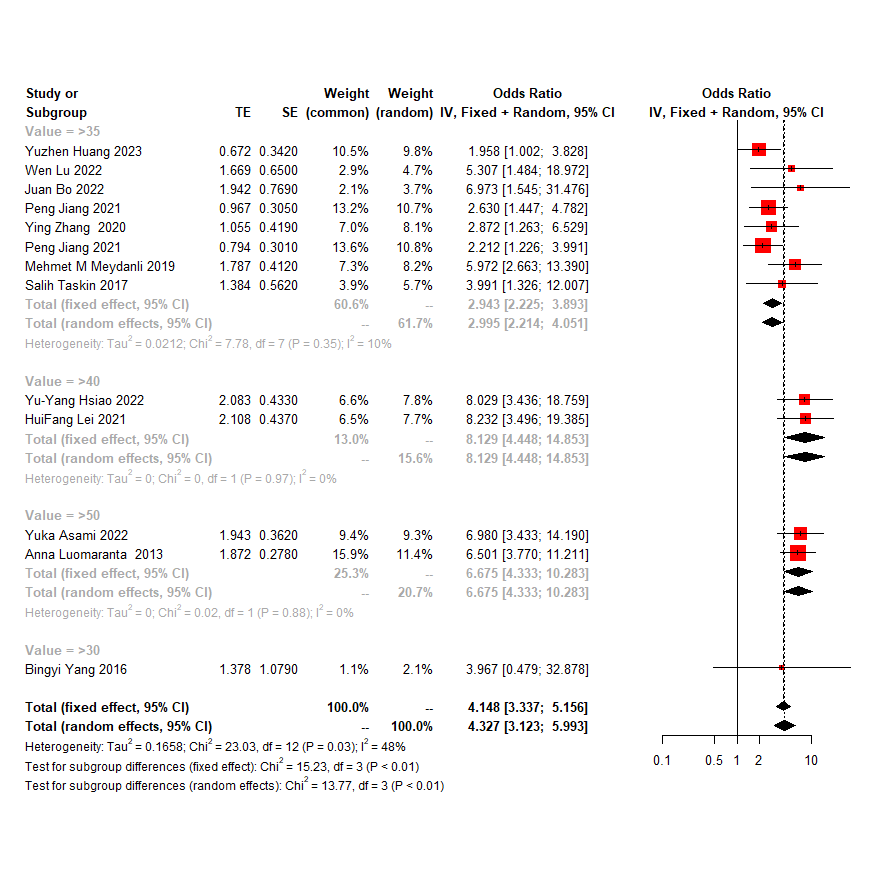


FigureS7 Forest plot of meta-analysis of CA125 for the prediction of endometrial carcinoma with lymph node metastasis


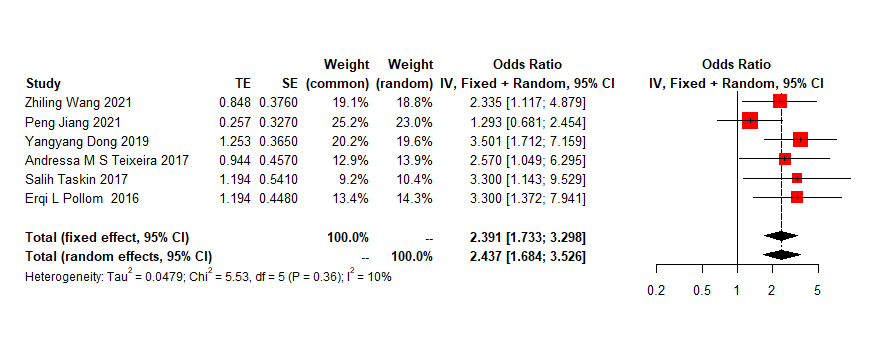


FigureS8 Forest plot of meta-analysis of Cervical Stromal Invasion for the prediction of endometrial carcinoma with lymph node metastasis


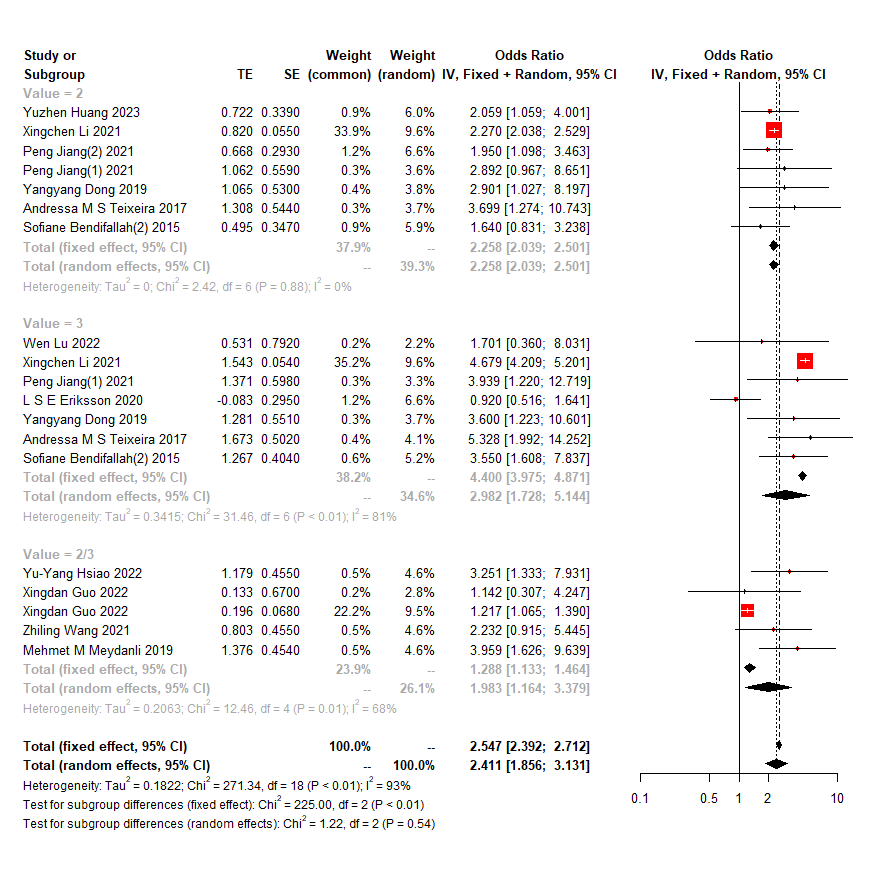


FigureS9 Forest plot of meta-analysis of Grade for the prediction of endometrial carcinoma with lymph node metastasis


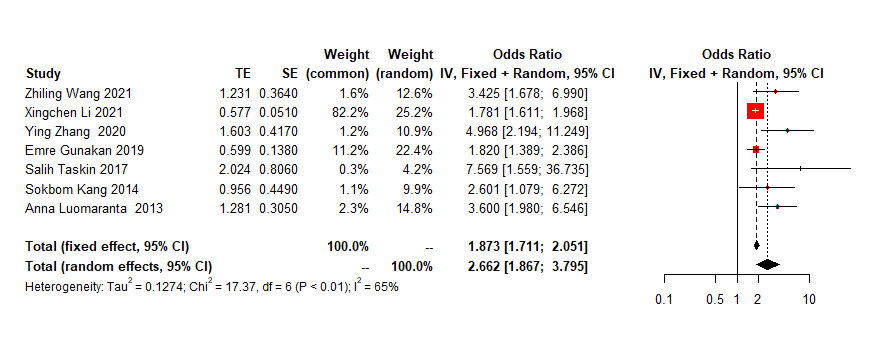


FigureS10 Forest plot of meta-analysis of Histological Type for the prediction of endometrial carcinoma with lymph node metastasis


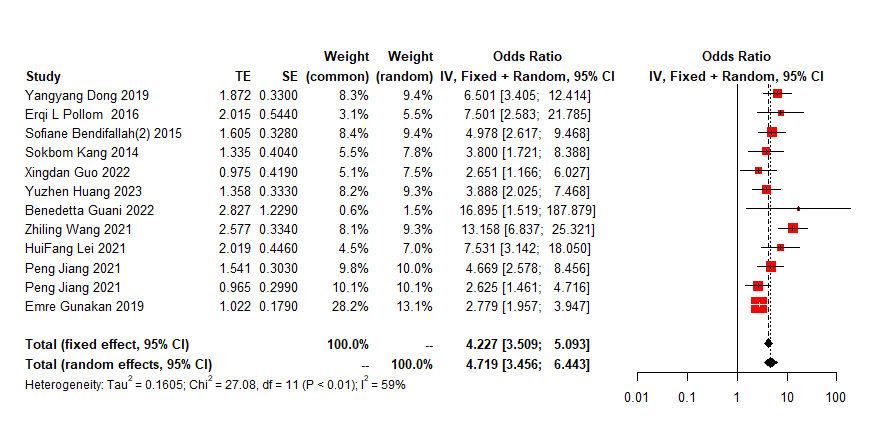


FigureS11 Forest plot of meta-analysis of Lymphatic Space Vascular Invasion for the prediction of endometrial carcinoma with lymph node metastasis


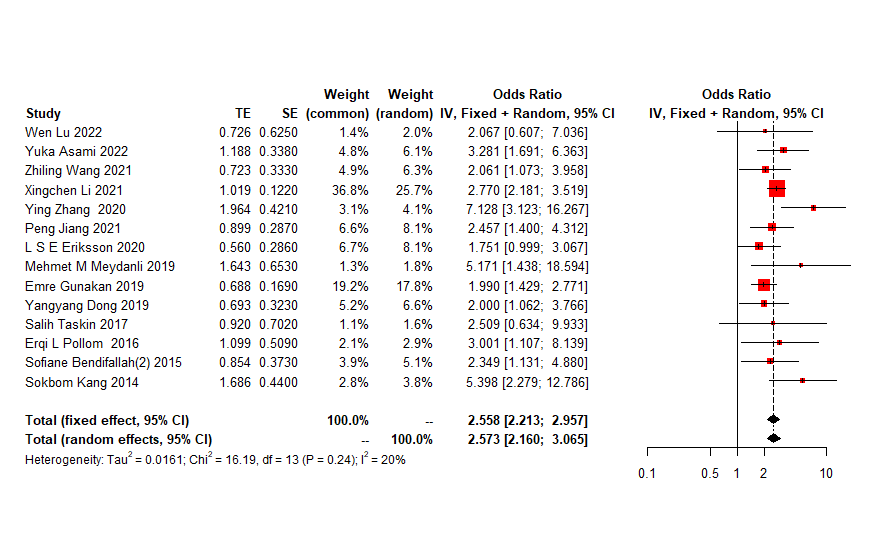


FigureS12 Forest plot of meta-analysis of Myometrial Invasion for the prediction of endometrial carcinoma with lymph node metastasis
